# Optimization of the microwave-assisted extraction of bioactive compounds from *Satureja hortensis* L.: an artificial neural network approach, chemical profile, extraction kinetics, and thermal properties

**DOI:** 10.3389/fnut.2025.1697165

**Published:** 2025-12-01

**Authors:** Darko Micić, Saša D. Đurović, Ivan Kojić, Angi E. Skhvediani, Yulia A. Smyatskaya, Yuri V. Shatalin, Tomislav Tosti, Julia G. Bazarnova, Jinhu Tian, Andrey V. Vasin

**Affiliations:** 1 Institute of General and Physical Chemistry, Belgrade, Serbia; 2 Institute of Biomedical Systems and Biotechnology, Peter the Great St. Petersburg Polytechnic University, Saint Petersburg, Russia; 3 Innovative Centre, Faculty of Chemistry, Belgrade, Ltd., University of Belgrade, Belgrade, Serbia; 4 Scientific Research Laboratory System Dynamics, Peter the Great St. Petersburg Polytechnic University, Saint Petersburg, Russia; 5 Institute of Theoretical and Experimental Biophysics, Russian Academy of Sciences, Pushchino, Russia; 6 Institute of Chemistry, Technology and Metallurgy, National Institute of the Republic of Serbia, University of Belgrade, Belgrade, Serbia; 7 College of Biosystems Engineering and Food Science, Zhejiang University, Hangzhou, China

**Keywords:** *Satureja hortensis*, microwave-assisted extraction, optimization, artificial neural network, chemical profile, biological activity, thermal properties, extraction kinetics

## Abstract

**Introduction:**

Summer savory is still not investigated thoroughly despite its potential and beneficial effects. Therefore, we aimed to investigate its chemical profile, thermal properties, extraction kinetics, and to optimize extraction process.

**Methods:**

Herein, an artificial neural network (ANN) was used as a nonlinear regression-based optimization model to optimize the microwave-assisted extraction of summer savory leaves. To achieve the goal, 17 experiments were conducted, combining different extraction times (20–40 min), ethanol concentrations (68–80%), and irradiation powers (400–800 W). Investigated responses included total phenolic content (TPC), total flavonoid content (TFC), DPPH, and ABTS assays. Kinetics was investigated by using four models, while thermal behavior was studied using DSC and TGA.

**Results:**

The highest outputs were: 256.36 mg GAE/g (GAE-gallic acid equivalents) (40 min, 40% ethanol, and 600 W) for TPC, 35.78 mg RU/g (rutin equivalents) (40 min, 60% ethanol, and 400 W) for TFC, 15.89 μg/mL (20 min, 60% ethanol, and 400 W) for DPPH, and 23.06 μg/mL (30 min, 60% ethanol, and 600 W) for ABTS. As a result of optimization, we obtained optimal extraction conditions (40 min, 52.8% ethanol, and 656.1 W) and predicted responses (246.50 mg GAE/g for TPC, 35.66 mg RU/g for TFC, and IC_50_ and EC_50_ values of 17.79 μg/mL and 25.79 μg mL for DPPH and ABTS assays, respectively). The experimentally obtained values for the investigated responses were 242.25 mg GAE/g, 36.30 mg RU/g, 17.10 μg/mL, and 24.48 μg/mL for TPC, TFC, IC_50_, and EC_50_, respectively. Total content of the quantified phenolic compounds was 91.47 μg/mL. The principal compound was rosmarinic acid (80.99 μg/mL), followed by chlorogenic acid (2.64 μg/mL), rutin (1.45 μg/mL), and apigenin (1.31 μg/mL).

**Discussion:**

The validity of the developed ANN model has been confirmed experimentally after preparing the extract under optimal conditions. Kinetic modeling showed that Model II provided the best fit for TFC, while Model IV provided the best fit for TPC. Optimally prepared extract showed high antioxidant activity and thermal behavior and could be used in food and pharmaceutical industry as an additive.

## 1 Introduction

The extraction of biologically active compounds from their natural sources and the application of the prepared extracts for various purposes have become a primary focus of researchers worldwide over the past several decades. Plants are recognized as the primary source of various classes of organic compounds, which are considered potentially beneficial for human health (1). One of these classes is phenolic and polyphenolic compounds, which are assigned a variety of biological activities, such as antioxidant, antimicrobial, cytotoxic, antiviral, and many others (2). Structurally, they can be polar, moderately polar, or non-polar. They occur in a bound form with carbohydrate moieties (such as glucose, rhamnose, or other) and in an aglycone (free) form as well (3, 4). The form that prevails in the extracts depends on the applied solvent, the extraction technique, and the extraction conditions (5–8). The widely explored activity of polyphenolic compounds is antioxidant activity. Their ability to counteract the actions of free radical species, i.e., reactive oxygen and reactive nitrogen species (ROS and RNS, respectively), makes them a natural defense against various disorders and diseases caused by ROS and RNS (2, 9, 10). Moreover, polyphenolic compounds are also being studied as possible substitutes for synthetic antioxidants, such as butylated hydroxyanisole (BHA) and butylated hydroxytoluene (BHT), due to their potentially harmless effects on the human body (11, 12).

It has been previously stated that plant material contains a wide range of compounds, each with distinct biological activities. One such plant is *Satureja hortensis* L. (summer savory), whose potential is not yet thoroughly investigated. It is an annual plant belonging to the *Lamiaceae* botanical family, specifically the genus *Satureja*. This plant is considered an aromatic, herbaceous shrub and is widely distributed in the Mediterranean and southern Europe (1, 13). The plant is known for its applications in both folk medicine and the food industry, as well as in various cuisines (14, 15). Medical applications include the treatment of cramps, nausea, indigestion, gastrointestinal disorders, and muscle pain (13, 16, 17). Furthermore, various biological activities are attributed to the plant’s parts and extracts. Among these are antioxidant, antiviral, antimicrobial, antifungal, sedative, antispasmodic, antinociceptive, hypoglycemic, hyperlipidemic, and antidiarrheal activities, as well as inhibition of amyloid beta protein aggregation (1, 11, 16–23). Regarding the chemical profile of the summer savory, different classes of compounds have been reported. The most important classes of compounds include terpenes, phenolic compounds, flavonoids, tannins, pyrocatechols, and steroids (24). Main compounds in the essential oil (terpenes) are carvacrol, γ-terpinene, thymol, and *p*-cymene (23, 25–28). Rosmarinic acid has been primarily reported as the main phenolic compound in summer savory (7, 11, 14, 19, 29, 30). Other phenolic compounds were found in much lower amounts in both glycosidic and aglycone forms (luteolin, catechin, rutin, epicatechin, hesperidin, and apigenin-7-glucoside) (7, 11, 30, 31). Given its monoterpenes, this plant has been used as a spice and for flavoring (13). It has been reported that summer savory is a good addition to chicken vinaigrette for turkey, goose, and duck. It can also be found in fricot and mince pies. It is also used for barbecues, stews, and sauces. Because of its rich aroma, summer savory is preferred for sausage applications over winter savory. Summer savory is also well known for its use in Bulgarian and Romanian cuisines (15).

Extraction techniques used to isolate natural compounds from plant material are divided into two major groups: conventional and non-conventional (32). Microwave-assisted extraction (MAE) is a non-conventional technique widely used for isolating bioactive compounds from natural sources. The heat is generated by interactions between microwaves and polar solvent molecules via ionic conduction and dipole-ion collisions (33–35). Various factors influence the MAE process and thereby significantly affect the yield of the compounds of interest. Among these factors are irradiation power, temperature, extraction time, heating cycle, solvent concentration, and solid-to-liquid ratio (SLR) (35). To obtain the maximum benefit from any extraction process, parameters must be carefully optimized. This makes the optimization process one of the most important steps in the process development.

Artificial neural networks (ANNs) are increasingly recognized as valuable tools for modeling and optimizing complex, nonlinear processes, particularly in the extraction of bioactive compounds from plant sources (36–38). Due to their ability to model multifactorial relationships without requiring explicit mathematical formulations, ANNs are well-suited for describing the intricate dynamics of extraction procedures. They can efficiently learn patterns from limited experimental datasets and provide nonlinear regression models that predict key outcomes, such as yield and antioxidant potential, across a wide range of operating conditions. When used in conjunction with optimization strategies, ANN-based modeling contributes to substantial reductions in experimental workload, offering a more streamlined and cost-effective approach to process development (39). Unlike traditional empirical approaches such as response surface methodology (RSM), which rely on predefined polynomial equations and assume limited interaction effects, ANN models can capture highly nonlinear and complex relationships between variables without such constraints. This flexibility allows for more accurate process description and supports the identification of near-optimal extraction conditions.

Lacking data on the optimization of summer savory extraction and a need for further information on the chemical profile and other properties of summer savory extracts, this study aimed to optimize the microwave-assisted extraction process of phenolic and polyphenolic compounds and antioxidant activity from summer savory using an artificial neural network. After optimization, the resulting model and conditions were used to prepare the extract. The prepared extract was analyzed for total phenolic and total flavonoid contents, as well as antioxidant activity, using two *in vitro* assays (DPPH and ABTS tests). The experimentally obtained values were then compared with the predicted ones. Furthermore, the extract prepared under optimal conditions was analyzed to establish a polyphenolic profile (UHPLC-DAD-MS/MS) and to obtain thermal behavior data (TGA and DSC). To the best of our knowledge, this is one of the rare studies on the thermal properties of the extract. Mainly, Đurović et al. (11, 40) reported the thermal properties of stinging nettle extracts and an ultrasound-assisted extract of summer savory.

## 2 Materials and methods

### 2.1 Plant material

Studied plant material (dried summer savory leaves) was purchased from the Institute “Dr. Josif Pančić” (Belgrade, Serbia) in 2022. The plant material was stored and prepared for extraction according to the previously described procedures (11). Briefly, plant material has been kept in paper bags and ground immediately before extraction.

### 2.2 Extraction procedures

The microwave-assisted extractions were done using the previously described equipment with a fixed liquid-to-solid ratio of 30 mL/g (6–8). Briefly, extractions were conducted using a domestic microwave oven adopted for this purpose (MM817ASM, Bosch, Germany). The irradiation power and extraction time were regulated using controllers on the oven. Investigated parameters were extraction time (20–40 min), ethanol concentration (40–80%), and microwave irradiation power (400–800 W). After the extraction, prepared extracts were filtered through filter paper to remove plant material and used for further analysis.

### 2.3 Optimization process

To model and optimize the microwave-assisted extraction (MAE) process of *Satureja hortensis* L., a computational nonlinear regression model inspired by an artificial neural network (ANN) structure was implemented. A total of 17 experimental runs were conducted, varying three key process parameters: extraction time (20, 30, and 40 min), ethanol concentration (40, 60, and 80%), and microwave power (400, 600, and 800 W). The measured responses were total phenolic content (TPC), total flavonoid content (TFC), and antioxidant activity indicators (IC₅₀ and EC₅₀). A multilayer perceptron (MLP) feedforward network trained via backpropagation was used to capture nonlinear relationships within the experimental design space. Computation was done with StatSoft Statistica (Version 12; StatSoft Inc., Tulsa, OK, United States). The dataset was randomly split into training (70%) and test (30%) sets. All input and output data were normalized using min-max scaling to ensure uniformity and improve model performance (39). To identify the optimal ANN architecture, a series of models was generated with hidden layer sizes ranging from 3 to 15 neurons. For each configuration, 10 independent networks were trained. The network with the best overall predictive performance, as measured by the highest coefficient of determination (*R*^2^), was selected. The performance of the selected ANN model was evaluated using several statistical metrics calculated jointly for the entire dataset (training and test combined). These included the coefficient of determination (*R*^2^), reduced chi-square (*χ*^2^), mean bias error (MBE), root mean square error (RMSE), and mean percentage error (MPE). All raw experimental data used for ANN training, including input parameters and measured responses for each of the 17 runs.

Yoon’s global sensitivity analysis was employed to determine the relative impact of each input variable (extraction time, ethanol concentration, and microwave power) on the output responses (TPC, TFC, IC₅₀, and EC₅₀). This method uses the ANN’s normalized weight coefficients to quantify the influence of variables across all hidden neurons and output nodes. The trained ANN model was also utilized as an interpolative tool for process optimization restricted to the experimental domain. Multi-objective optimization was performed using the Solver tool in Microsoft Excel (Version 2019; Microsoft Corporation, Redmond, WA, United States), aiming to maximize TPC and TFC while minimizing IC₅₀ and EC₅₀. This integrative modeling and optimization approach enabled the identification of optimal extraction conditions using the trained network, enhancing process efficiency without requiring additional experimental trials. It should be noted that the model is intended for interpolation within the tested conditions and is not a fully generalizable predictive ANN due to the limited dataset size.

### 2.4 Total phenolic and flavonoid contents

The total phenolic content (TPC) and total flavonoid content (TFC) were estimated using a well-established, previously described procedure (41, 42). The tests rely on the reaction of the compounds in the extract with the reagents, producing colored solutions whose absorbance was measured at specific wavelengths.

The TPC was determined according to the following procedure: the reaction mixture was prepared by mixing 0.1 mL of the extract, 7.9 mL of distilled water, 0.5 mL of Folin–Ciocalteu reagent, and 1.5 mL of sodium carbonate (20%, w/w). After incubation at room temperature for 1 h, absorbance was measured at 750 nm. The blank was prepared by replacing the extract with distilled water. The total phenolic content of the obtained extracts was calculated by interpolating the measured sample absorbance into a calibration curve defined with standard solutions of gallic acid.

For the determination of the TFC, the extract was mixed with 5% NaNO_2_ solution (0.3 mL). After 5 min, aluminum chloride hexahydrate (10%, 0.3 mL) was added, and the mixture was allowed to stand for 6 min. Sodium hydroxide (1 mol/dm^3^, 1 mL) was added to the mixture, and distilled water was added to bring the final volume to 10 mL. The blank was prepared by replacing the extract with distilled water. Immediately after mixing, absorbance was measured at 510 nm. The TPC was calculated by interpolating the measured sample absorbance from a calibration curve generated with rutin standard solutions.

All measurements were done in triplicate. The final results are expressed as the milligrams of gallic acid equivalents (mg GAE/g) and milligrams of rutin equivalents (mg RU/g) per gram of the extract.

### 2.5 Antioxidant activity

The antioxidant activity was assessed using two *in vitro* spectrophotometric methods: DPPH radical and ABTS radical scavenging assays. The DPPH assay was performed using a previously established procedure (43) with minor adaptations (44). Briefly, the extracts were diluted with methanol and mixed with a solution of 2,2-diphenyl-1-picrylhydrazyl (DPPH) radical in 95% methanol. The absorbance of the mixture was measured at 515 nm after a 60-min incubation at room temperature. Blank solution was prepared by adding ethyl acetate instead of the sample. The results were expressed as IC_50_ values (μg/mL), which means the amount of the sample required to neutralize 50% of radical species. The IC_50_ values were obtained by constructing a curve plotting RSC (radical scavenging activity) as a percentage against the concentration of the extract. The %RSC was calculated from the following equation:


$$\%\mathrm{RSC}=100-\frac{A_{1}\times 100}{A_{2}}$$

where *A*_1_ is the absorbance of the sample solution and *A*_2_ is the absorbance of the blank solution.

The ABTS assay is also a widely used, well-established, and previously described method (45). Briefly, 38.4 mg of ABTS and 6.6 mg potassium persulfate (K_2_S_2_O_8_) were dissolved in 10 mL of water and stored in the dark for 16 h. After formation of the stable radical cation (ABTS^•+^), the solution was diluted with ethanol to a concentration with UV absorption of 0.70 ± 0.20 at 734 nm. Then, 990 μL of diluted ABTS solution was mixed with 10 μL of the analyzed extract and left at room temperature for 20 min. Results were calculated using the same equation as for the DPPH assay. The results are expressed as EC_50_ in μg/mL, while the tests for all analyzed samples in both assays were performed in triplicate.

### 2.6 Kinetics evaluation

The kinetic evaluation of the MAE extracts prepared under the optimal conditions was assessed with four empirical models. Experimentally obtained data for the TPC and TFC were used for the assessments. The models were the first-order rate law model (Model I), Peleg’s hyperbolic model (Model II), Elovich’s equation (Model III), and the power-law model (Model IV), represented by the following equations:


(Model I) $$C_{t}=C_{\mathrm{eq}}(1-e^{-k_{1}t})$$

(Model II) $$C_{t}=\frac{C_{\mathrm{eq}}t}{k_{2}^{\prime }+t}$$

(Model III) $$C_{t}=\mathrm{Eln}(t)+a$$

(Model IV) $$C_{t}=Bt^{n}$$

All the above-named models are previously elaborated and explained (46). In all four equations, *C_t_* (mg/mL) and *t* (min) were obtained experimentally and represent the dependent and independent variables, respectively. All other parameters were calculated accordingly (46).

### 2.7 Analysis of the phenolic and polyphenolic compounds

The chemical profile of the extract prepared under optimal conditions was assessed using a Dionex Ultimate 3,000 UHPLC equipped with a diode array detector (DAD) and a TSQ Quantum Access Max mass spectrometer (Thermo Fisher Scientific, United States). Operational parameters of the used method, used column, and other details regarding the method were previously described in detail (47, 48). The separation process was performed at 40 °C on a Syncronis C18 column (100 × 2.1 mm, 1.7 μm particle size) from Thermo Fisher Scientific. The mobile phase consisted of 0.01% acetic acid in water (A) and acetonitrile (B), and used in the following gradient elution: 5% B in the first 2 min, 2nd–12th minutes 5–95% B, 12th–13th minutes from 95 to 5% B, and 5% B until the 20th minute. The flow rate was set to 0.3 mL/min, and the detection wavelengths to 254 and 280 nm. The injection volume was 5 μL.

Stock methanolic solutions of polyphenolics in a concentration of 1,000 mg/L were prepared. The stock solutions were mixed and diluted with water in order to obtain working solutions (concentrations of 0.01, 0.05, 0.10, 0.25, 0.50, 0.75, and 1.00 mg/L). A TSQ Quantum Access Max triple-quadrupole mass spectrometer equipped with heated electrospray ionization (HESI) source was used with the vaporizer temperature kept at 250 °C, and ion source settings as follows: spray voltage 4,500 V, sheet gas (N2) pressure 27 AU, ion sweep gas pressure 0 AU and auxiliary gas (N2) pressure 7 AU, capillary temperature 275 °C, skimmer offset 0 V, and capillary offset −35 V. The mass spectrometry data were acquired in negative ionization mode, over the *m*/*z* range of 100 to 1,000. Multiple mass spectrometric scanning modes, including full scan (FS) and product ion scan (PIS), were used for qualitative analysis of the targeted compounds. The collision-induced fragmentation experiments were performed using argon as the collision gas, with the collision energy varying depending on the compound. Time-selected reaction monitoring (tSRM) experiments for quantitative analysis were performed using two MS2 fragments for each compound, previously identified as dominant in the PIS experiments. Xcalibur software (version 2.2) was used for instrument control. Phenolics were identified and quantified based on their spectral characteristics: molecular ions, mass spectra, characteristic fragmentations, and characteristic retention times. The limits of detection (LOD) and quantification (LOQ) were calculated using the standard deviation (SD) of the responses and the slope (S) of the calibration curves according to: LOD = 3 × SD/S and LOQ = 10 × SD/S. The results of the analysis are given as micrograms of the compounds per milliliter of the liquid extract (μg/mL).

### 2.8 Thermal properties

The thermal behavior of the dried summer savory extract was assessed using Thermogravimetric Analysis (TGA) and Differential Scanning Calorimetry (DSC) on a TA Instruments equipment (TGA Q500 and DSC Q1000, Delaware, United States). For TGA, approximately 7.0 ± 0.5 mg of the sample was heated from room temperature to 600 °C at 5 °C/min under a nitrogen atmosphere with a flow rate of 60 mL/min. For DSC analysis, approximately 3.0 ± 0.5 mg of the sample was placed in a hermetically sealed aluminum pan, while an identical, sealed, empty pan was used as the reference. The measurements were carried out over the temperature range of 10–120 °C at a heating rate of 5 °C/min under a nitrogen flow of 50 mL/min. The obtained data were analyzed using TA Advantage Universal Analysis 2000 software.

### 2.9 Statistical analysis

All analyses were performed in triplicate, and the data are presented as mean values with standard deviations (±SD). Statistical differences among group means were evaluated using one-way analysis of variance (ANOVA), followed by Tukey’s honest significant difference (HSD) *post hoc* test at a significance threshold of *p* < 0.05. Statistical comparison between experimental and predicted (ANN and kinetic models) values was performed using a paired t-test to evaluate the significance of differences (*p* < 0.05). The statistical evaluation was performed using XLSTAT software (version 2014.5.03, Addinsoft, New York, United States), integrated within Microsoft Excel.

## 3 Results and discussion

### 3.1 Total phenolics and flavonoids content

The summarized results for the TPC and TFC are given in Table 1. The TPC ranged from 91.28 mg GAE/g (20 min, 80% ethanol, and 600 W) to 256.36 mg GAE/g (40 min, 40% ethanol, and 600 W). The TFC ranged from 15.75 mg RU/g (20 min, 80% ethanol, and 600 W) to 35.78 mg RU/g (40 min, 60% ethanol, and 400 W). The results presented herein indicated that the lowest values were obtained under the same conditions. Also, a high ethanol percentage was obviously not suitable for these classes of compounds (flavonoids could be considered a subclass of the phenolic compound class). The same extraction time (40 min) yielded the maximum amount of these compounds. However, a higher ethanol concentration was more suitable for flavonoid isolation (60% for TFC and 40% for TPC), while a higher irradiation power was more suitable for TPC (600 W). Differences in the solubility of phenolics and flavonoids are conditioned by their chemical structures, which further influence their polarity and solubility in different solvents. The lower polarity of flavonoids compared to other phenolic compounds in the extracts required a higher ethanol concentration in the solvent. Moreover, not only phenolic compounds react with the Folin reagent in the TPC assay. Many other classes react with this regent. Among them are carbohydrates, amino acids, nucleotides, thiols, unsaturated fatty acids, proteins, vitamins, amines, aldehydes, and ketones (6, 49). Therefore, the TPC is not selective toward phenolic compounds, and the reactivity of many other classes leads to significant discrepancies between TPC and HPLC results (5, 7, 8). Furthermore, the TFC content decreased with increasing irradiation power, indicating the molecules’ sensitivity to temperature and microwaves.

**Table 1:** Effect of extraction parameters on TPC, TFC, IC₅₀, and EC₅₀ during microwave-assisted extraction (MAE).

Independent variables			Measured responses			
Time (min)	Conc. ethanol (%)	Power (W)	TPC (mg GAE/g)	TFC (mg RU/g)	IC_50_ (μg/mL)	EC_50_ (μg/mL)
20	40	600	238.77 ± 7.65^a^	33.54 ± 1.65^ab^	20.05 ± 0.60^gh^	32.34 ± 0.96^bcde^
20	60	400	167.27 ± 3.42^c^	33.69 ± 1.49^ab^	15.89 ± 0.41^i^	28.74 ± 1.36^fgh^
20	80	600	91.28 ± 2.31^d^	15.75 ± 0.74^d^	26.26 ± 0.84^de^	33.80 ± 0.95^abcd^
20	60	800	168.40 ± 6.63^c^	31.51 ± 1.27^b^	18.42 ± 0.53^h^	26.72 ± 1.24^ghi^
30	40	400	242.61 ± 7.63^a^	34.11 ± 0.73^ab^	22.00 ± 0.56^fg^	35.68 ± 1.66^ab^
30	40	800	240.74 ± 6.28^a^	33.63 ± 1.33^ab^	35.00 ± 1.65^a^	30.64 ± 0.75^def^
30	60	600	190.62 ± 9.20^b^	33.78 ± 1.35^ab^	18.74 ± 0.64^h^	25.48 ± 0.89^hij^
30	60	600	191.97 ± 6.03^b^	35.16 ± 0.97^a^	18.58 ± 0.87^h^	23.58 ± 0.87^ij^
30	60	600	189.35 ± 8.00^b^	33.10 ± 0.72^ab^	18.53 ± 0.47^h^	23.06 ± 0.89^j^
30	60	600	196.09 ± 5.80^b^	35.60 ± 1.11^a^	18.63 ± 0.59^h^	27.00 ± 1.12^ghi^
30	60	600	189.35 ± 5.59^b^	34.68 ± 0.89^ab^	20.26 ± 0.77^gh^	25.30 ± 1.04^hij^
30	80	400	93.12 ± 2.76^d^	18.32 ± 0.42^cd^	29.11 ± 0.71^c^	31.54 ± 1.33^def^
30	80	800	97.27 ± 3.65^d^	17.39 ± 0.82^cd^	31.74 ± 0.89^b^	36.48 ± 1.58^a^
40	40	600	256.36 ± 7.92^a^	35.07 ± 0.95^a^	28.47 ± 0.96^cd^	35.06 ± 1.05^abc^
40	60	400	176.69 ± 8.44^bc^	35.78 ± 1.03^a^	19.16 ± 0.48^h^	29.64 ± 1.00^efg^
40	60	800	194.22 ± 8.11^b^	35.11 ± 0.98^a^	24.21 ± 0.92^ef^	26.04 ± 1.04^hij^
40	80	600	108.87 ± 2.83^d^	20.08 ± 0.57^c^	28.37 ± 0.84^cd^	31.90 ± 1.26^cdef^

The values are presented as mean ± SD (*n* = 3), different superscripts within the same column indicate significant differences of means, according to Tukey’s HSD test (*p* < 0.05).

The IC_50_ and EC_50_ refer to the antioxidant activity.

Different research groups studied this plant and its phytochemicals. However, several articles in the literature reported varying content of these compounds. Reported diversities originate from the differences in factors that influence plant growth and thus secondary metabolism (e.g., geographical origin, seasonal variations, environmental factors, soil composition, and many others) (11). The other reason is the way the results are expressed. Namely, different equivalents have been used to express the total phenolics and flavonoids contents in the analyzed extracts. Despite the differences, we will correlate and compare the results of the previously conducted studies and draw some conclusions.

Al-Juhaimi et al. (50) studied the TPC and TFC in the hydrosols obtained during and after the hydrodistillation. The authors reported TPC and TFC in the ranges of 362.30–442.46 mg/L and 21.43–35.71 mg/L, respectively. Samples were drawn after 1, 60, and 120 min. The results showed that both TPC and TFC were higher in the sample obtained after 1 min than in the sample obtained after 60 min. In both cases, the highest yield was observed in the sample after 120 min. Optimization of the ultrasound-assisted extraction (UAE) using the response surface methodology (RSM) provided very insightful information about the process and influence of the operational parameters (ethanol concentration, temperature, and liquid–solid ratio) on the selected responses (total phenolics content, total flavonoid content, DPPH radical scavenging activity, and ABTS radical scavenging activity) (11, 30). Mašković et al. (30) reported the TPC and TFC in ranges of 3.36–4.40 mg CAE/g and 1.79–2.23 mg RE/g, respectively (CAE-chlorogenic acid equivalents and RE-rutin equivalents). Đurović et al. (11) used gallic acid equivalents (GAE) and rutin equivalents (RU) to express the TPC and TFC in prepared extracts. The authors reported TPC and TFC in ranges of 110.88–145.88 mg GAE/g and 19.69–24.53 mg RU/g, respectively. Besides the differences in the results, there were differences in the reported optimal conditions for UAE. One group reported a 20% ethanol concentration, 80 °C, and a solid–liquid ratio, while another group reported a 40% ethanol concentration, 80 °C, and a solid–liquid ratio.

A previous study conducted by Mašković et al. (7) compared different extraction techniques (Soxhlet extraction, maceration, ultrasound-assisted extraction, microwave-assisted extraction, and subcritical water extraction) for the isolation of phenolics and flavonoids from summer savory leaves. The authors found that subcritical water extract (SWE) had the highest TPC and TFC contents and was the most potent extract, followed by MAE and UAE. The reported TPC and TFC for MAE were 147.21 mg GAE/g and 23.10 mg RU/g, respectively. Both results are consistent with those obtained in this study, where the values reported by Mašković et al. (7) are higher than the lowest TPC and TFC values and lower than the highest TPC and TFC values in this study. After extracting the TPC from summer savory using the Soxhlet apparatus with ethanol and acetone, Exarchou et al. (19) reported TPCs of 6,400 mg and 21,500 mg of caffeic acid equivalents per 100 g of extract in the ethanolic and acetone extracts, respectively. Results indicated that acetone was a better solvent for the isolation of phenolic compounds, contrary to the findings of Zeković et al. (6), who reported lower performance of acetone compared with ethanol. Alonso-Alonso-Carrillo et al. (2) also studied different extraction techniques, including heat reflux (HRE) and microwave-ultrasound-assisted extraction (MUAE), for the isolation of phenolic and flavonoid compounds from the *Satureja* plant. The authors reported TPC of 96.91–141.69 mg GAE/g and 91.01–166.09 mg GAE/g for HRE and MUAE, respectively (2). The same authors also reported the TFC content in the same extracts, ranging from 52.61 to 112.22 mg Cat/g and 30.65 to 123.88 mg Cat/g (milligrams of catechin equivalents per gram of extract). The combination of MAE and UAE was superior to heat reflux for the isolation of phenolics and flavonoids (2). Interestingly, the lowest TPC and TFC values for MUAE were observed in the water extract, while the highest contents were reported in 75% ethanol (TPC) and 100% ethanol (TFC). However, a preliminary study of the UAE extract from stinging nettle leaves indicated water was superior as a solvent compared with 96% ethanol (6). Absolute (100%) ethanol was a better choice for isolating flavonoid compounds in both techniques (HRE and MUAE). In contrast, for the TPC, 50% ethanol yielded the best result in HRE, whereas 75% ethanol was a better choice for MUAE (2).

### 3.2 Antioxidant activity

The results of the antioxidant assays are summarized in Table 1. The DPPH assay (IC_50_ values) showed activity in the range of 15.89–35.00 μg/mL, while the ABTS assay (EC_50_ values) showed activity ranging from 23.06 μg/mL to 36.48 μg/mL. In this particular case, a higher value indicates lower activity because IC_50_ and EC_50_ values represent the amount of the extract required to neutralize 50% of the existing radical species in the solution. The highest activity against DPPH and ABTS radicals was obtained with extracts prepared under the following conditions: 20 min, 60% ethanol, and 400 W (DPPH); and 30 min, 60% ethanol, and 600 W (ABTS). The 60% ethanolic solution was the best choice for the solvent in both cases. Activity against the ABTS radicals required longer extraction and higher irradiation power than for the DPPH. These differences in activity could be explained by the distinct properties of the radical species and the varying mechanisms of action in these assays. Namely, the DPPH radicals are not soluble in water and are only slightly soluble in most organic solvents. At the same time, the ABTS is a cation that is soluble in both water and organic solvents, thereby increasing its ability to react with various antioxidant agents. Furthermore, it was reported that the structural conformation of the natural compounds plays an important role in determining their reactivity toward DPPH (11, 51).

A search of the existing literature also revealed diversity in the reported activities. The main reasons are the same as for the diversity in the TPC and TFC, i.e., geographical origin, differences in chemical composition due to variations in environmental factors, seasonal differences, and the extraction technique, solvents, and conditions used to prepare the extracts. Exarchou et al. (19) reported activities of 95.8 and 33.0% for the ethanolic and acetone extracts, respectively. This confirmed the substantial impact of the selected solvent on the composition and, consequently, on the extract’s activity. Moreover, the same authors also reported that the acetone extract showed higher TPC than the ethanolic extract, implying that phenolic compounds are not the only compounds responsible for the antioxidant activity. The ethanolic macerate also showed activity against the ABTS radical species (2.44 μg/mL) and hydrogen peroxide (28.04 μg/mL) (16). Mašković et al. (7) also compared the activity of the extracts prepared with conventional and non-conventional extraction techniques. Authors reported SWE (23.33 μg/mL) as the most potent against the DPPH radical species, followed by the MAE (32.87 μg/mL). Mašković et al. (30) and Đurović et al. (11) optimized the UAE using RSM, studying the influence of operational parameters on the DPPH and ADTS assays as responses. Reported activities against DPPH radicals were in the range of 0.020–0.047 mg/mL and 18.18–42-73 μg/mL. The EC_50_ values for the ABTS assays were 0.029–0.041 mg/mL (30) and 26.36–39.09 μg/mL (11). Comparing the activity of the extracts in this study, we observed that MAE produced an extract with higher activity than UAE, confirming previously reported findings from different groups comparing the efficiency of various extraction techniques (5–8, 40, 52). In addition to the TPC and TFC, Alonso-Carrillo et al. (2) reported the activities of HRE and MUAE against DPPH and ABTS radical species. The authors found that the most potent HRE extract against the DPPH and ABTS radicals was obtained using 50% ethanol after 120 min of extraction. On the other hand, the MUAE extract obtained using 100% ethanol after 30 min of extraction was the most potent against ABTS. In comparison, 50% ethanol and 120 min of extraction gave the extract with the best performance against DPPH radical species. The displayed diversities in antioxidant activity confirmed the previous discussion regarding the significance and influence of operational conditions on the chemical profile and, therefore, the activity of the prepared extracts.

### 3.3 Optimization of the MAE

The architecture of the ANN-based nonlinear regression model proved to be a key factor in achieving accurate predictive performance, with particular emphasis on the number of neurons in the hidden layer and the initialization strategy for weights and biases. Given the sensitivity of ANN training to these initial conditions, a systematic procedure was employed to enhance model robustness and reduce stochastic variability. The number of neurons in the hidden layer was incrementally varied from 3 to 15. For each configuration, 10 training runs were performed with randomly initialized weights and biases, yielding 130 unique ANN models. Of the total models developed, 126 achieved a coefficient of determination (*R*^2^) above 0.85, reflecting consistently strong predictive accuracy across the tested configurations. The overall mean *R*^2^ value was 0.948, confirming the ANN’s capacity to model the complex nonlinear relationships between the input variables (extraction time, ethanol concentration, and microwave power) and the target outputs (TPC, TFC, IC_50_, and EC_50_) within the limited experimental dataset.

**Figure 1:**
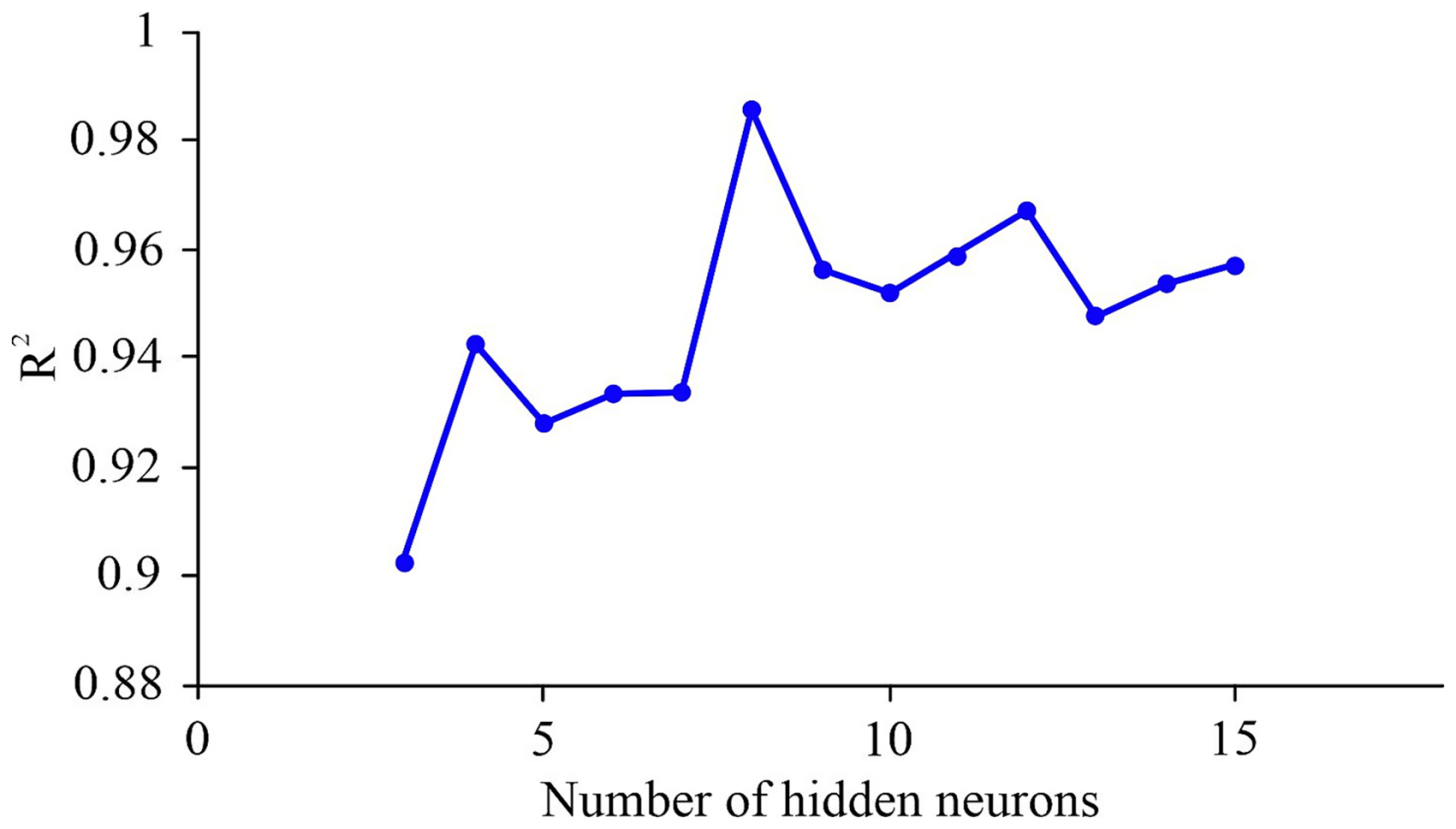
Influence of hidden neurons number on *R*^2^ mean value.

As illustrated in Figure 1, the average *R*^2^ values increased with the number of hidden neurons, peaking at eight; beyond this point, additional neurons did not yield notable gains in accuracy. All tested models showed average *R*^2^ values exceeding 0.90, underscoring the model’s stability and learning efficiency. The optimal network, with eight hidden-layer neurons (MLP 3-8-4, Table 2), achieved an *R*^2^ of 0.98 and was selected for further optimization. This architecture provided an optimal balance between model complexity and predictive fit, while the limited dataset constrains the model’s generalizability and prevents full extrapolation beyond the studied parameter range.

**Table 2:** Artificial neural network model summary (performance and errors) for training and testing cycles.

Network name	Performance		Error		Hidden activation	Output activation
	Training	Test	Training	Test		
MLP 3-8-4	0.985	0.980	46.977	56.20	Tanh	Logistic
	Training algorithm		Error function			
	BFGS 30		SOS			

**Figure 2:**
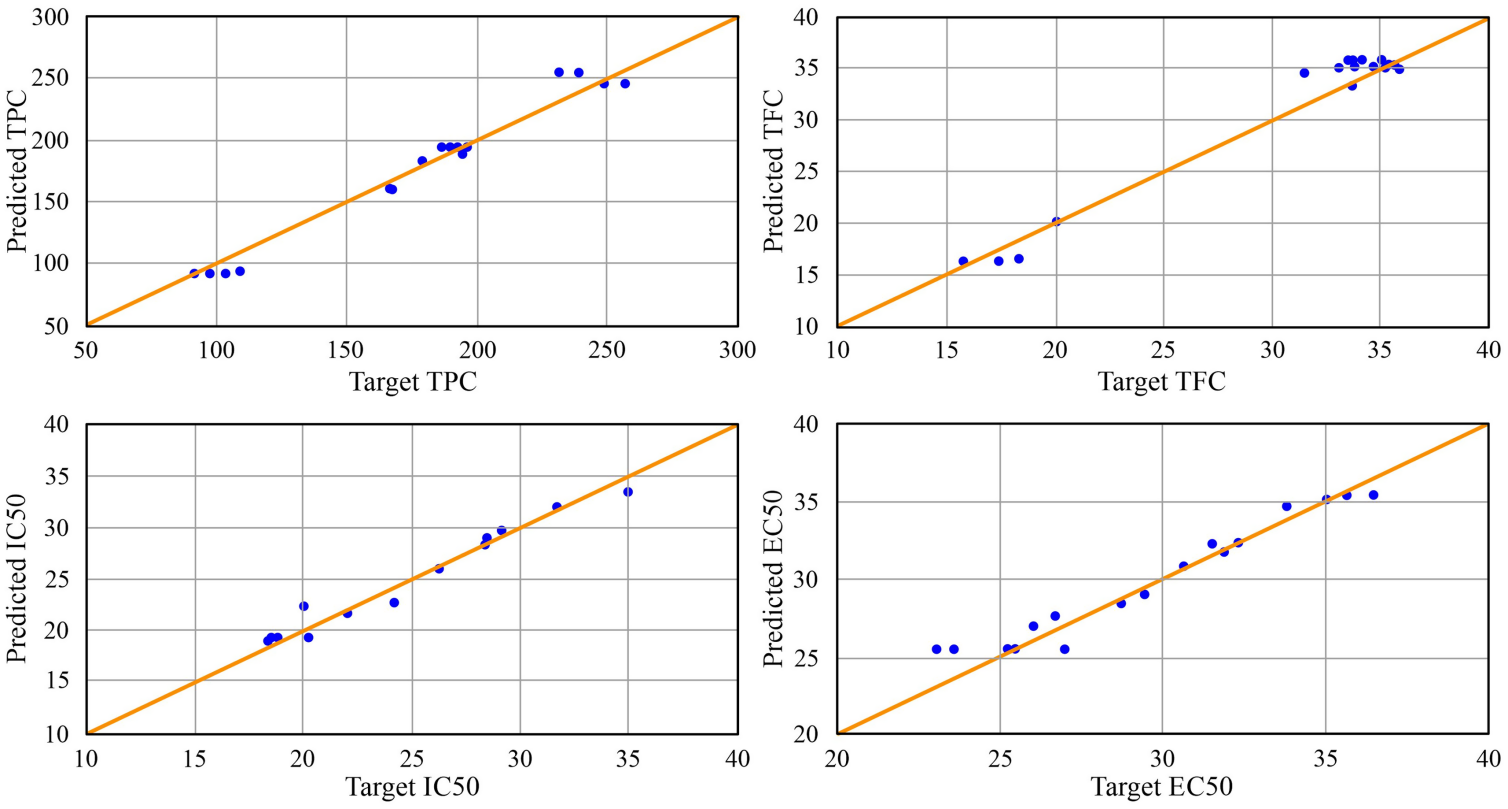
Experimental and predicted values obtained for TPC, TFC, IC_50_ and EC_50_.

The developed model demonstrated solid predictive performance across the tested experimental conditions. As shown in Figure 2, the predicted values aligned closely with the experimental measurements, indicating that the model adequately captured the underlying system behavior within the tested domain. No statistically significant differences (*p* > 0.05) were observed between experimental and ANN-predicted values (Table 3), confirming the high predictive accuracy of the developed model. Also, the performance metrics presented in Table 3 support this observation. Coefficients of determination (*R*^2^) for TPC, TFC, IC_50_, and EC_50_ were 0.976, 0.975, 0.965, and 0.953, respectively, suggesting a consistent and reliable fit between the model and experimental data. Additional validation using error indicators showed that the model maintained acceptable accuracy. Mean percent error (MPE) ranged from 2.620 to 4.608%, while root mean square error (RMSE) values were between 0.987 and 9.761. The mean bias error (MBE) was close to zero in all cases (ranging from −0.088 to −0.576), indicating a low level of systematic deviation. Analysis of residuals further confirmed the model’s robustness. The distribution of residuals, as indicated by skewness and kurtosis, did not exhibit significant asymmetry or heavy tails, suggesting a generally well-behaved prediction error structure.

**Table 3:** The “goodness of fit” tests for the developed ANN model.

	*χ* ^2^	RMSE	MBE	MPE	*r* ^2^	Skew	Kurt	Mean	StDev	Var	Mean (exp)	Mean (pred)	*p*
TPC	101.238	9.761	−0.277	4.608	0.976	−0.819	0.774	−0.277	10.058	101.157	178.5053	178.7825	0.91095
TFC	2.177	1.431	−0.576	3.876	0.975	−0.142	−0.631	−0.576	1.351	1.825	30.38127	30.95679	0.098142
IC50	1.144	1.038	−0.088	3.907	0.965	0.464	0.433	−0.088	1.066	1.136	23.14241	23.2307	0.737154
EC50	1.034	0.987	−0.281	2.620	0.953	−0.505	0.685	−0.281	0.975	0.951	29.57765	29.85821	0.252754

**Figure 3:**
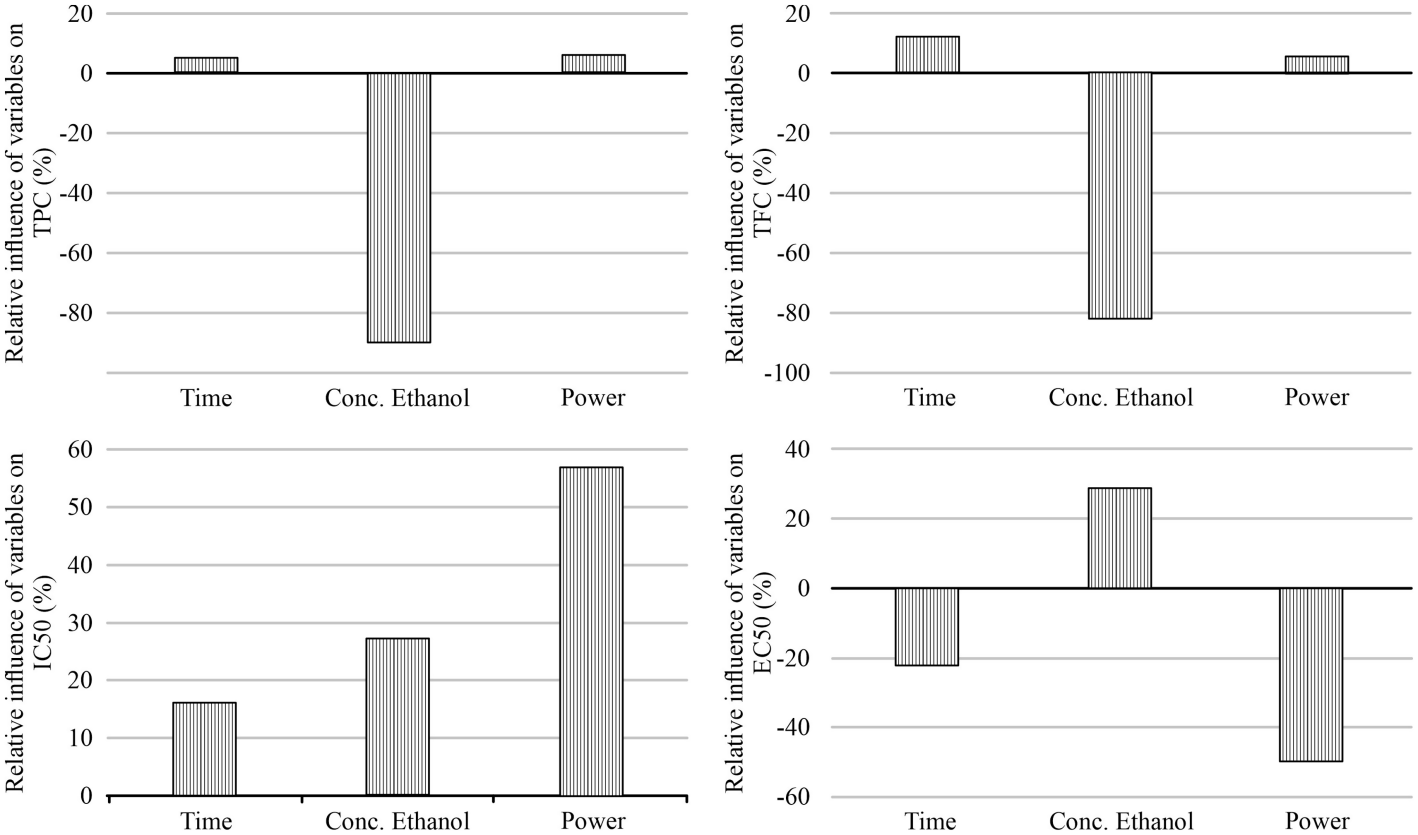
The relative importance of the time, concentration of ethanol and power on TPC, TFC, IC_50_ and EC_50_.

Yoon’s global sensitivity analysis provided insight into the relative influence of the extraction parameters (extraction time, ethanol concentration, and microwave power) on the predicted values of TPC, TFC, IC_50_, and EC_50_. As shown in Figure 3, ethanol concentration exhibited the most pronounced negative influence on both TPC (−89.72%) and TFC (−82.86%), indicating that increasing ethanol content reduced the extraction efficiency of polyphenolic and flavonoid compounds, potentially due to the lower solubility of more polar bioactives in highly ethanolic media. In contrast, extraction time (+4.64% for TPC; +12.04% for TFC) and microwave power (+5.64% for TPC; +5.10% for TFC) had a comparatively minor yet positive influence on these responses, indicating that their increase generally enhanced the extraction yield within the tested range.

A different trend was observed for the antioxidant activity indicators. For IC_50_, microwave power (+56.84%) had the most substantial positive effect, followed by ethanol concentration (+27.03%) and extraction time (+16.12%). Since lower IC_50_ values correspond to higher antioxidant activity, this positive influence indicates that increasing these parameters raised the numerical IC_50_ values, thereby reducing antioxidant potential—a likely consequence of partial degradation of thermolabile compounds at higher energy input. For EC_50_, the effects of microwave power (−49.63%) and extraction time (−22.03%) were negative, indicating that increasing these parameters reduced EC_50_ values and thereby enhanced antioxidant activity. Conversely, ethanol concentration showed a moderate positive influence (+28.35%), suggesting that higher ethanol content slightly increased EC_50_, consistent with a lower antioxidant capacity. These apparent discrepancies between IC_50_ and EC_50_ trends arise from differences in the reaction mechanisms and kinetics of the two antioxidant assays. Overall, the analysis emphasizes that the impact of individual parameters varies with the target response, underscoring the importance of balanced process optimization to efficiently extract the desired bioactive compounds.

Optimization of the MAE process was conducted to determine the most effective combination of extraction parameters for maximizing the yield of bioactive compounds and antioxidant activity. The optimization procedure employed the Solver tool in Microsoft Excel and was based on the developed regression-type ANN model. The research was restricted to the experimentally investigated ranges of extraction time (20–40 min), ethanol concentration (60–80%), and microwave power (400–800 W).

The ANN model predicted that optimal extraction conditions (40.0 min, 52.8% ethanol concentration, and irradiation power of 656.1 W) would result in the following outcomes: 246.50 mg GAE/g for TPC, 35.66 mg RU/g for TFC, and IC_50_ and EC_50_ values of 17.79 μg/mL and 25.79 μg/mL, respectively (Table 4). These results indicate favorable extraction performance across polyphenol and flavonoid yields and antioxidant potential. Notably, the optimal ethanol concentration lies near the center of the tested range, suggesting that a moderate solvent polarity was beneficial for the simultaneous extraction of both phenolics and flavonoids. Experimental validation (Table 4) under these optimized conditions yielded results that closely matched the predicted values, supporting the model’s reliability for interpolation within the tested range. It should be noted, however, that the small dataset limits the ANN model’s predictive power, and extrapolation beyond the tested conditions should be interpreted cautiously. The model is best considered as a tool for trend analysis and correlation-based optimization within the experimental domain, rather than a fully generalizable machine learning predictor.

**Table 4:** Predicted and experimental values under the optimized MAE conditions determined by ANN (40.0 min, 52.8% ethanol, 656.1 W).

Values	Investigated responses			
	TPC (mg GAE/g)	TFC (mg RU/g)	IC_50_ (μg/mL)	EC_50_ (μg/mL)
Predicted	246.50	35.66	17.79	25.79
Experimental	242.25	36.30	17.10	24.48

### 3.4 Chemical profile of optimized extract

The chemical characterization of the optimized extracts is provided by TPC, TFC, and UHPLC analyses of extracts prepared under optimal conditions (Tables 4, 5). The optimal values for TPC, TFC, IC_50_, and EC_50_ were 246.50 mg GAE/g, 35.66 mg RU/g, 17.79 μg/mL, and 25.79 μg/mL, respectively (Table 4). To verify the model’s validity and the optimization results, we prepared an extract under optimal conditions. We obtained values of 242.25 mg GAE/g, 36.30 mg RU/g, 17.10 μg/mL, and 24.48 μg/mL for TPC, TFC, IC_50_, and EC_50_, respectively (Table 4). Comparing the predicted and experimental values for all four responses in Table 4, it may be concluded that the model is valid and that the optimization was successful. Moreover, comparing the optimal MAE values herein with previously published results of RSM optimization for UAE by Mašković et al. (11) and Đurović et al. (30), the MAE technique was again more efficient, providing an extract with higher TPC and better antioxidant activity as well.

**Table 5:** Phenolic profile of *Satureja hortensis* L. extract prepared under optimal conditions.

Compound (RT^a^)	Content (μg/mL)
*Phenolic acids*	
*p*-Hydroxybenzoic acid (5.09)	0.32
Caffeic acid (5.48)	0.14
Rosmarinic acid (6.79)	80.99
Chlorogenic acid (5.04)	2.64
Syringic acid (5.88)	0.19
*p*-Coumaric acid (6.25)	0.12
Ferulic acid (6.70)	0.39
Sinapic acid (5.67)	0.13
*Coumarins*	
Aesculin (Esculetin 6-β-D-glucoside) (4.28)	0.21
Aesculetin (5.82)	0.31
*Flavonoids*	
Rutin (quercetin-3-*O*-rutinoside) (5.94)	1.45
Apigenin-7-*O*-glucoside (6.83)	0.70
Quercetin (7.80)	0.32
Quercetin-3-*O*-glucoside (6.91)	0.30
Quercetin-3-rhamnoside (6.75)	0.15
Luteolin (7.75)	0.71
Naringenin (7.92)	0.17
Naringin (naringenin-7-*O*-neohesperidoside) (6.15)	0.23
Kaempferol (8.57)	0.72
Apigenin (8.44)	1.31
Summary	91.47

a RT-retention time (min).

**Figure 4:**
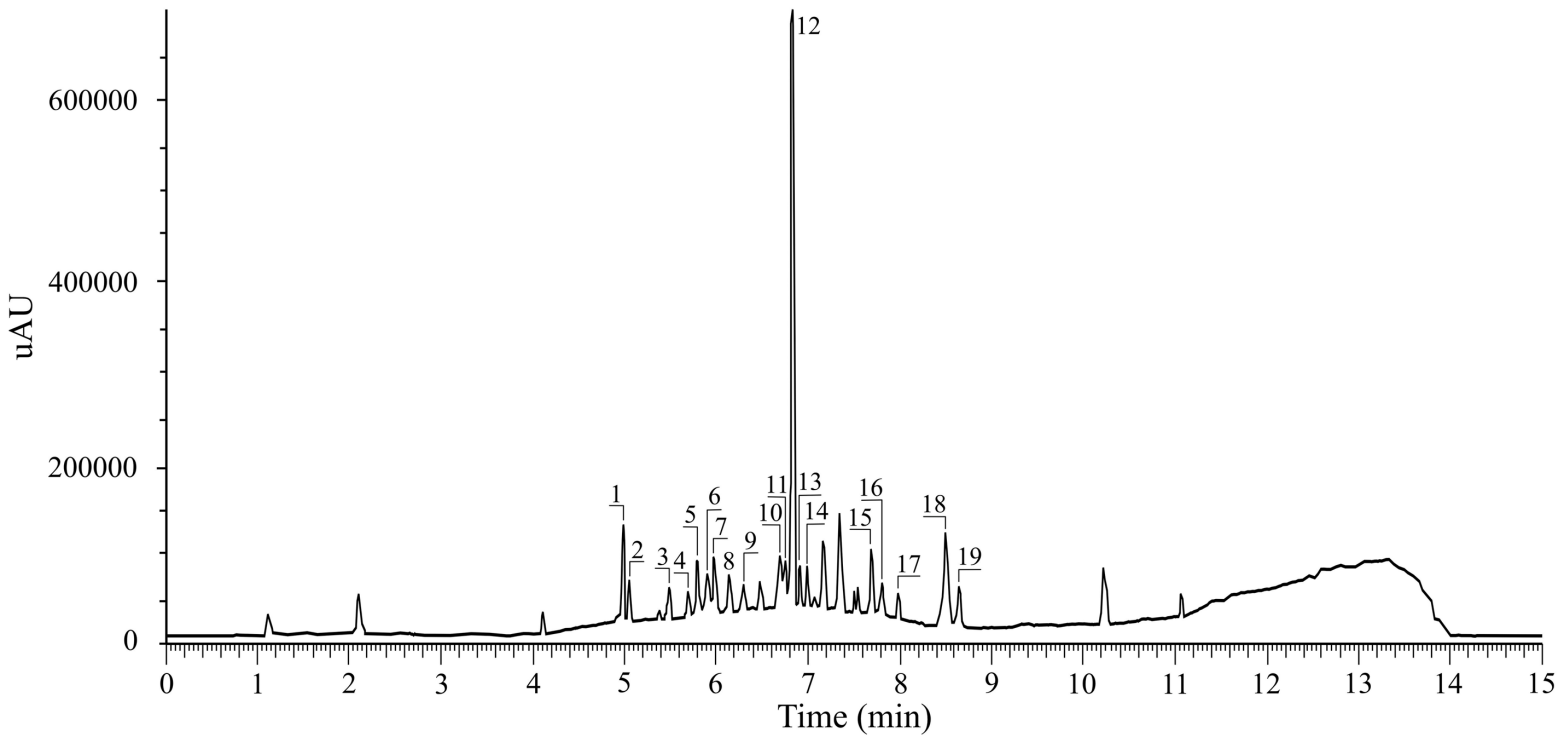
HPLC chromatogram of *Satureja hortensis* L. extract: (1) Chlorogenic acid, (2) *p*-Hydroxybenzoic acid, (3) Caffeic acid, (4) Sinapic acid, (5) Aesculetin, (6) Syringic acid, (7) Rutin (quercetin-3-*O*-rutinoside), (8) Naringin (naringenin-7-*O*-neohesperidoside), (9) *p*-Coumaric acid, (10) Ferulic acid, (11) Quercetin-3-rhamnoside, (12) Rosmarinic acid, (13) Apigenin-7-*O*-glucoside, (14) Quercetin-3-*O*-glucoside, (15) Luteolin, (16) Quercetin, (17) Naringenin, (18) Apigenin, (19) Kaempferol.

The chemical profile of the extract obtained under optimal conditions is summarized in Table 5, and the chromatogram is shown in Figure 4. The total content of the quantified phenolic compounds was 91.47 μg/mL, while the principal compound was rosmarinic acid with the content of 80.99 μg/mL, which comprises 88.5% of the total content. The other three compounds with higher concentrations were chlorogenic acid, rutin, and apigenin, at 2.64 μg/mL, 1.45 μg/mL, and 1.31 μg/mL, respectively. Detected compounds may be classified as phenolic acids and polyphenolic compounds. Polyphenolic compounds could be divided into coumarins and flavonoids. Compounds from both groups can be glycosides (carbohydrate-containing compounds) or aglycones (the free forms). A common form in nature is the glycosidic form. However, during the extraction, hydrolysis of the glycosides occurred, and free forms (aglycones) were later detected in the extracts (3, 4, 47, 48). Considering that we use a mixture of water as a solvent (in combination with ethanol) and microwave irradiation, which provides energy for various reactions, we detected both forms herein. Thus, we have aesculin and aesculetin, apigenin-7-*O*-glucoside and apigenin, quercetin and its glucoside and rhamnoside, and naringin and naringenin (Table 5).

The presence of phenolic and polyphenolic compounds in the analyzed extract is significant, given the wide range of biological activities ascribed to them: antioxidant, cardioprotective, anticarcinogenic, anti-inflammatory, antimicrobial, and many others (9). Regarding the presence and activity of the individual compounds, *p*-hydroxybenzoic acid is known for its antioxidant, antimicrobial, and estrogenic activities (53). Caffeic acid, which is present in many vegetables, exists as a monomer, dimer, trimer, or as a derivative attached to the flavonoids (9). Its primary role is to protect plants against pests and to provide UV protection (54). It shows primary and secondary antioxidant activity, where the primary represents the ability to react with radical species, while the secondary represents the ability to act as a chelating agent, forming a complex that further prevents decomposition of the peroxide and the formation of free radical species (55). The complex compounds with copper are reported to be helpful in cancer treatment because of the pro-oxidant ability to damage cancer cells by inducing lipid peroxidation (56). Ferulic acid is a derivative of caffeic acid, and its activity is similar to that of the latter. Different biological activities have been ascribed to ferulic acid. Among them are antimicrobial, anti-diabetic, anti-inflammatory, anti-thrombotic, vasodilatory, hepatoprotective, and anti-carcinogenic (9). Aesculin is also commonly present in many plants. It has been known for its antibacterial, antioxidant, anti-inflammatory, anticarcinogenic, and anti-arteriosclerotic activities. It has been reported that this compound increases the activity of superoxide dismutase and glutathione and suppresses dopamine-dependent ROS overproduction in human neuroblastoma cells (9, 57). Its anti-inflammatory activity is reflected in the suppression of expression of the inflammatory factors, such as inducible nitric oxide synthase, interleukin-1β, and tumor necrosis factor-α (58). It has also been reported that aesculin inhibits the proliferation of carcinoma cells by interfering with the mitochondrial apoptosis pathway (57) and decreases triglyceride levels and inhibits the proliferation of vascular soft muscle (59). Naringin has been reported to be a potent anti-inflammatory agent and suppressor of interleukin-6, nitric oxide, nitric oxide synthesis, and tumor necrosis factor-α production (60). Moreover, naringin delays tumor growth *in vivo*, increases phosphorylation of AMP-activated protein kinase, and inhibits cell growth (61). Quercetin has also been reported as an effective anti-inflammatory compound. It inhibits inflammatory factors like tumor necrosis factor-α and interleukin-1α (62). Due to its antioxidant activity, quercetin can suppress the proliferation of cancer cells. This activity is achieved through the reduction of oxidative stress and the suppression of multiple kinase proteins responsible for cancer (63).

Previously published studies on the chemical profile of summer savory extracts also named rosmarinic acids as the principal compound (13, 19). Boroja et al. (13) also reported caffeic acid as the second most abundant compound in the extracts, but Exarchou et al. (19) reported it at trace levels (0.09 μg/mL). Naringenin, apigenin, and isoferulic acid were also reported in significant amounts. We did not detect isoferulic acid in our extract. Mašković et al. (7) also reported high levels of rosmarinic acid (9.62 μg/mL), the third most abundant compound in the MAE extract, after quercetin (41.26 μg/mL) and rutin (28.48 μg/mL). Interestingly, rosmarinic acid was the most abundant in the extract prepared with conventional extraction techniques (maceration and Soxhlet extraction). At the same time, rutin and quercetin had the highest yield in UAE, MAE, and SWE. An optimal extract prepared after UAE optimization showed the opposite results. Both authors found rosmarinic acid at significantly higher concentrations (46.172 μg/mL and 52.25 μg/mL) than all other detected compounds (11, 30). Moreover, Mašković et al. (11) did not report the presence of *p*-hydroxybenzoic acid, caffeic acid, sinapic acid, syringic acid, and naringenin in their extract, whereas we found these compounds in both the UAE and MAE extracts reported herein. Al-Juhaimi et al. (50) investigated the chemical profile of hydrosols obtained using hydrodistillation. They reported kaempferol (2.10–19.95 mg/L), catechin (12.86–216.47 mg/L), and rutin (1.35–9.49 mg/L) as the main compounds. Results showed that after 1 min of extraction, kaempferol was the principal compound. After 60 min, catechin was the principal compound, followed by kaempferol, while after 120 min, kaempferol was the fourth main compound after catechin, rutin, and gallic acid, other compounds reported in higher amounts were gallic acid (1.61–4.00 mg/L), 3,4-dihydroxybenzoic acid (1.67–3.89 mg/L), caffeic acid (0.76–1.52 mg/L), and quercetin (0.84–1.08 mg/L). Surprisingly, authors did not report the presence of rosmarinic acid in any of the analyzed hydrosols (50).

### 3.5 Thermal properties of the optimized extract

**Figure 5:**
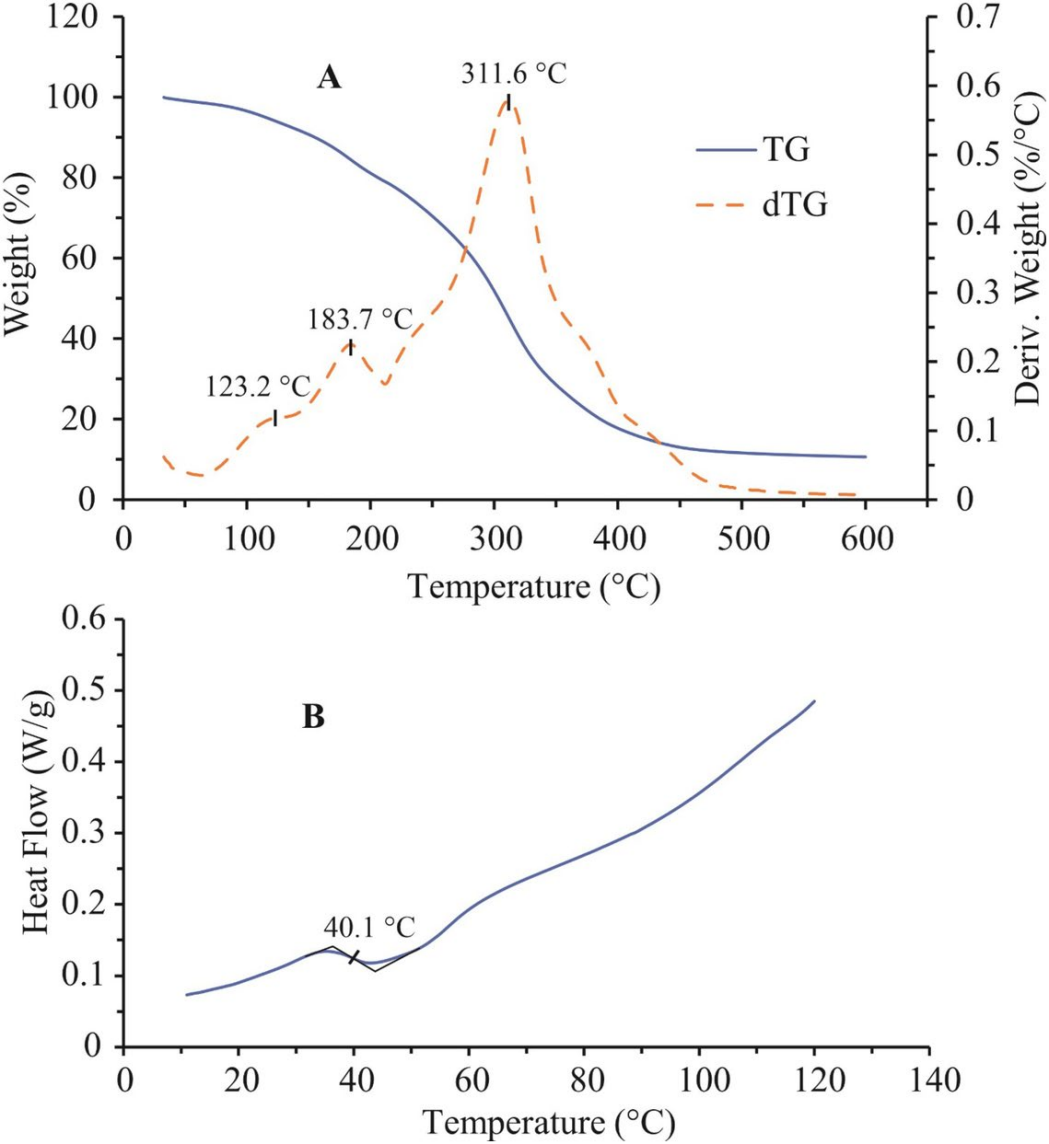
**(A)** TG/dTG and **(B)** DSC thermograms of dried summer savory extract. Heating rate 5 °C/min.

Thermal profiles of the dried summer savory extract are presented in Figure 5. Analysis of the thermogravimetric (TG) and derivative thermogravimetric (dTG) curves (Figure 5A) indicates that the thermal decomposition of the sample in the range from room temperature to 600 °C proceeds through three distinct stages, as evidenced by the three peaks observed on the dTG curve. The first decomposition step, occurring between room temperature and 135 °C with a peak temperature (Tp) of 123.2 °C, is associated with a weight loss of approximately 6.9%. This stage reflects the release of moisture and low-molecular-weight volatile compounds present in the extract. The second stage, observed between 135 and 210 °C, corresponds to a Tp at 183.7 °C and is accompanied by a 14.1% mass loss. This phase marks the initiation of thermal degradation of organic and bioactive constituents in the extract. Given the sensitivity of these components to elevated temperatures, careful thermal control within this range is crucial to preserve the extract’s functional properties. The third degradation step, occurring between 210 and 500 °C with a peak at 311.6 °C, accounts for the most substantial weight loss (67.4%). The broad nature of this peak, along with shoulders on both sides, suggests the complexity of the thermal decomposition process in this temperature range. Initially, thermally stable macromolecules that resisted decomposition in earlier phases start to degrade. As the temperature rises further, secondary reactions such as the polymerization of degradation intermediates and their subsequent breakdown become increasingly prominent. These overlapping processes contribute to the complex appearance of the dTG signal in this region (40). At the end of the heating process, approximately 10.6% of the sample mass remained as residue at 600 °C. The overall thermal stability observed up to approximately 180 °C suggests that the extract can withstand moderate processing temperatures, which is advantageous for its incorporation into food matrices, spray-drying, or encapsulation systems. Above this threshold, significant degradation of phenolic compounds likely occurs, indicating that processing operations such as baking or high-temperature drying should be carefully controlled to minimize thermal losses.

DSC analysis identified a glass transition (Tg) of the dried summer savory extract at approximately 40.1 °C (Figure 5B). A similar thermal behavior has been reported for dried nettle extract, which exhibited a glass transition around 37 °C (40), indicating that this class of plant extracts may possess comparable physicochemical characteristics. The observed glass transition temperature is particularly relevant, as it marks the onset of increased molecular mobility within the amorphous matrix of the dried extract. This parameter is critical for understanding the material’s thermal and physical stability, especially during storage and processing. Maintaining the extract below its Tg is essential to prevent undesired changes such as caking, stickiness, or loss of bioactive compound stability, thereby ensuring the retention of functional properties in food or pharmaceutical formulations (64). From a practical standpoint, the relatively low Tg indicates that the extract is prone to physical transitions at ambient conditions, which may affect its handling and shelf stability. Therefore, encapsulation into protective carriers or storage under controlled humidity and temperature conditions could enhance its stability. In food preservation or nutraceutical formulations, these findings highlight the importance of maintaining the amorphous extract below Tg to preserve antioxidant potency and prevent structural collapse during long-term storage.

### 3.6 Kinetics modelling of the optimized extract

**Figure 6:**
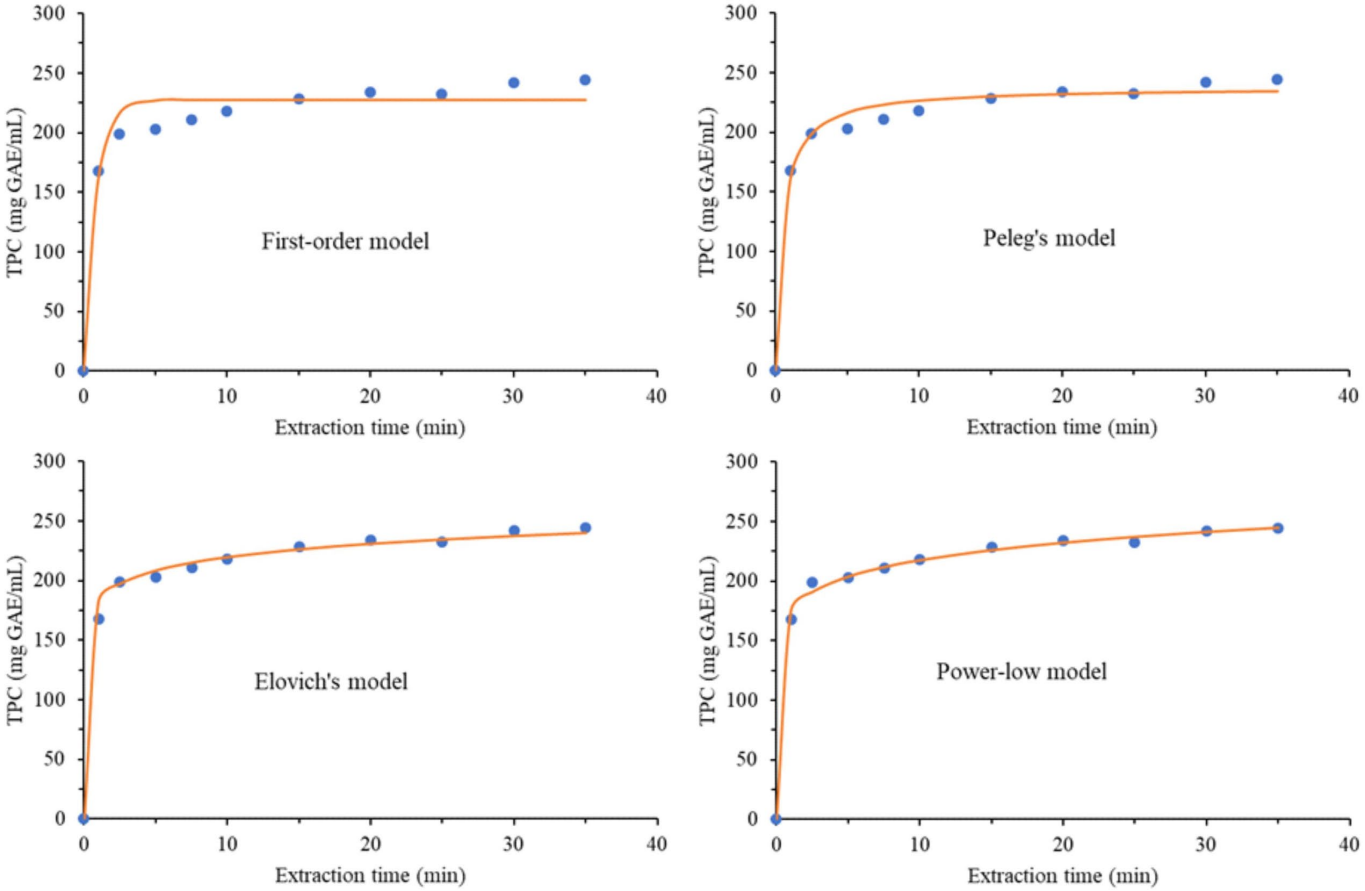
Experimentally obtained data and fitting curves of TPC for investigated kinetics models.

**Figure 7:**
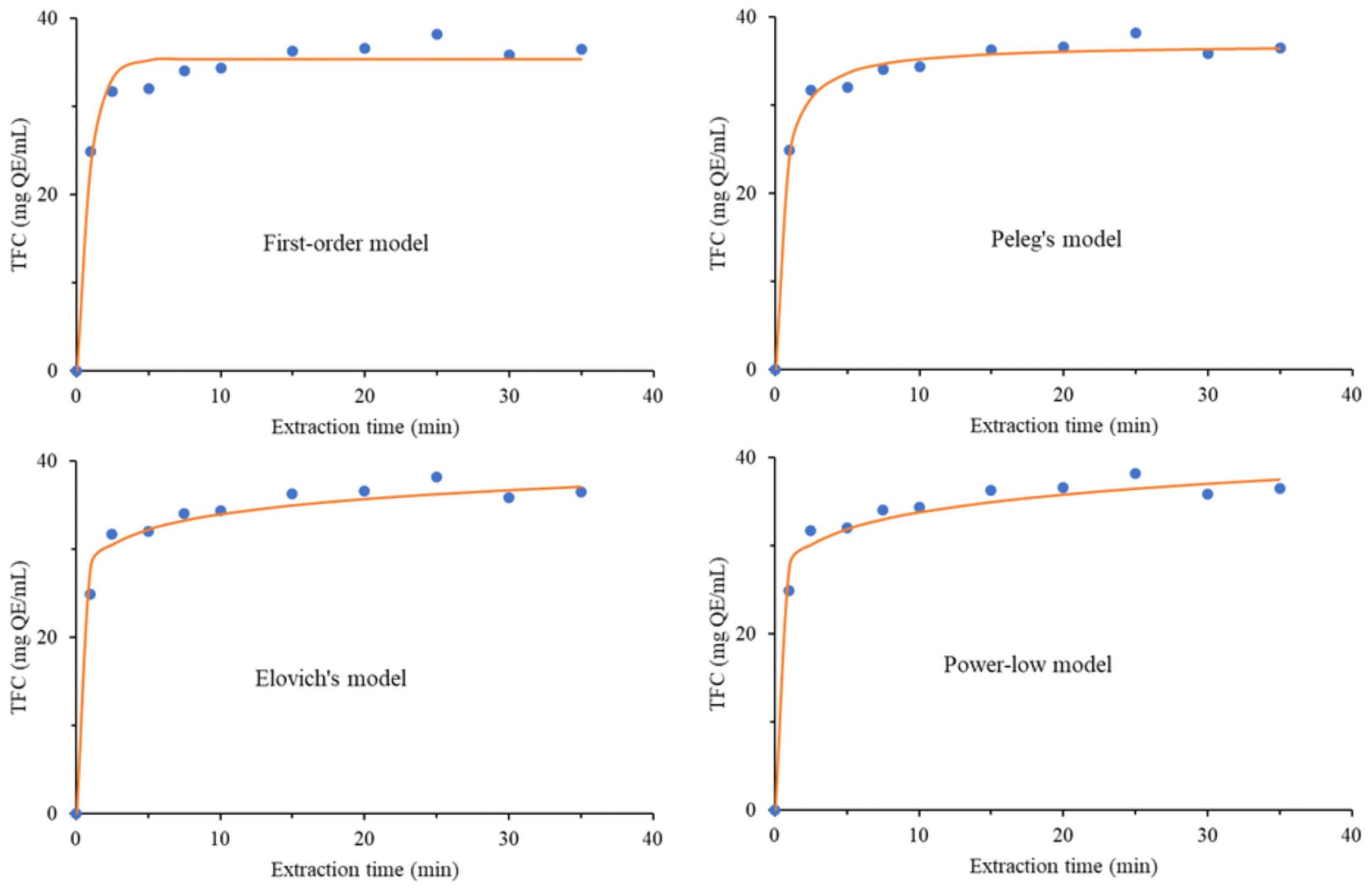
Experimentally obtained data and fitting curves of TFC for investigated kinetics models.

The kinetics of the extraction process were evaluated for the extract prepared under optimal conditions for TPC and TFC by fitting the data to the four empirical models described by the equations provided in Table 6, which also includes calculated parameters for both evaluated parameters (TPC and TFC). According to the paired t-test, there was no significant difference between the experimental and predicted TPC and TFC values across all models (*p* > 0.05), confirming the models’ accuracy. Also, the sum of squared errors (SS_er_), the coefficient of determination (*R*^2^), and the average absolute relative deviation (AARD) describe the accordance between the experimentally obtained values and the used models. The *R*^2^ values for all four models were higher than 0.950 for both TPC and TFC. The SS_er_ values were particularly high, except for Model II for TFC. Generally, this parameter was substantially higher for TPC across all four models, whereas it was significantly lower for TFC. The ARRD values were lower than 5% except for Model I for TPC. Therefore, Model IV provided the best fit for the TPC, while Model II provided the best fit for TFC. The kinetic curves for all four models are shown in Figures 6, 7.

**Table 6:** Calculated parameters and statistical data of four empirical models applied for MAE kinetics of total phenols (TPC) and flavonoid (TFC) content.

Model equation		Model parameters						Statistical parameters					
		*k*	*C*_eq_	*E*	*a*	*B*	*n*	*R* ^2^	SS_er_	AARD (%)	Mean (exp)	Mean (pred)	*p*
Model I	TPC	1.21	227.6					0.956	2,220	5.7	202.12	202.00	0.976
	TFC	1.13	35.4					0.976	28.9	4.0	31.35	31.34	0.973
Model II	TPC	0.49	237.9					0.984	778	3.2	202.12	202.06	0.980
	TFC	0.49	36.9					0.991	9.9	2.2	31.35	31.35	0.994
Model III	TPC			16.2	182.1			0.993	357	2.2	202.12	202.12	0.999
	TFC			2.49	28.3			0.979	23.9	3.7	31.35	31.35	0.999
Model IV	TPC					174.9	0.09	0.997	159	1.3	202.12	202.13	0.994
	TFC					27.9	0.08	0.978	25.8	4.1	31.35	31.36	0.992

The most influential parameters in MAE are solvent polarity, solvent concentration, irradiation power, sample nature and properties, solvent-to-sample ratio, temperature, extraction time, and mixing type and speed (65). All these parameters will influence TPC and TFC to some extent. In our case, we examined the effects of irradiation power, extraction time, and solvent concentration on TPC and TFC (Table 1). Previous studies on the kinetics of MAE and the influence of parameters have shown that irradiation has a positive effect up to a certain power, after which TPC decreases (46). The optimal irradiation power in our study was a value that needed to be investigated (600 W) rather than the maximum value (800 W). A similar result was obtained for ethanol concentration. However, the optimal value for the extraction time was maximal (40 min). The negative influence of irradiation power was attributed to the initiation of different reactions among the compounds and their subsequent degradation (66). The negative influence of irradiation power was also confirmed in the extraction from *Morus nigra* fruits, albeit with a minor effect compared to other parameters, such as ethanol concentration and time (67). Pavlić et al. (46) also reported an adverse effect of high irradiation power on polyphenolic extraction from peppermint, concluding that the optimal irradiation power is 600 W, which is consistent with our finding.

Regarding extraction time, it was reported that extending the extraction process may lead to decomposition of the essential oil constituents (68). Moreover, prolonged exposure to microwave irradiation may also degrade polyphenolic compounds (69). Interestingly, optimal extraction time herein was 40 min. Considering that we obtained an extract with high TPC, TFC, and antioxidant activity, we can conclude that degradation did not occur; instead, structural transformation occurred, creating more potent compounds that consequently increased the extract’s antioxidant activity (5–8). Thus, the occurrence of Maillard reactions was reported as a consequence of exposure to microwave irradiation, which consequently produces new, more potent compounds (70–72).

Careful analysis of the kinetic parameters in Table 6 reveals a correlation between the rate constant (*k*) and the saturation concentration (*C*_eq_). The rate constants for TPC and TFC in Model I were similar, whereas they were the same in Model II. The saturation concentrations for TPC and TFC in Models I, II were also almost the same. The parameters power-law exponent (*n*) and extraction rate constant (*B*) are adjustable parameters, where *n* < 1, which was actually the case. Parameter *B* provided information on the solvent-compound system during the extraction process. Therefore, the irradiation power interacts with both the solvent and the sample. The concentration gradient decreased sharply due to rapid extraction of the compounds at the beginning of the process, resulting in a lower diffusion exponent at higher irradiation power. Parameters *a* in Model II and *B* in Model IV are also very similar. Pavlić et al. (46) reported that the first-order model (Model I) was the best solution for describing the kinetics of MAE extraction of polyphenolic compounds for peppermint. However, in our case, Models II, IV were better choices for TFC and TPC, respectively.

## 4 Conclusion

To optimize microwave-assisted extraction of summer savory leaves, 17 experiments were conducted with five repetitions at central points. An artificial neural network (ANN) model was used for optimization, with responses including TPC, TFC, DPPH, and ABTS assays. The ANN model successfully predicted the conditions and values of the responses, as confirmed experimentally by preparing and analyzing an extract under the predicted optimal conditions. Optimal conditions were 40 min, a 52.8% ethanol solution, and an irradiation power of 656.1 W. The predicted values were 246.50 mg GAE/g for TPC, 35.66 mg RU/g for TFC, and IC₅₀ and EC₅₀ values of 17.79 μg/mL and 25.79 μg/mL, respectively. Experimentally measured values for the same responses were 246.50 mg GAE/g, 35.66 mg RU/g, 17.79 μg/mL, and 25.79 μg/mL. The close agreement between predicted and experimental results validated the ANN-based regression model’s reliability for process optimization within the studied range.

The optimal extract was also analyzed using UHPL-DAD-MS/MS to assess its phenolic and polyphenolic profile. The most abundant compound was rosmarinic acid, followed by chlorogenic acid, rutin, and apigenin. Thermal properties were studied using TGA and DSC. The results showed that the extract’s decomposition proceeded through three distinct stages. The first stage occurred between room temperature and 135 °C, reflecting the release of moisture and low-molecular-weight volatile compounds. The second stage, observed between 135 °C and 210 °C, corresponds to the initiation of thermal degradation of the organic and bioactive constituents in the extract. The third degradation step occurred between 210 °C and 500 °C and accounted for the most substantial weight loss (67.4%).

Investigation of extraction kinetics revealed that Model II provided the best fit for TFC, whereas Model IV provided the best fit for TPC. This study also indicated that prolonged extraction times and elevated irradiation powers may lead to decomposition and/or side reactions, altering the chemical profile of the extract.

## Data Availability

The raw data supporting the conclusions of this article will be made available by the authors, without undue reservation.
